# Should Voluntary and Poor-Law Hospitals Combine?

**Published:** 1920-09-25

**Authors:** 


### The Hospital

The Workers' Newspaper of Administrative Medicine and Institutional Life, Administration, National Insurance and Health.

No. 1790, Vol. LXVIII. SATURDAY, SEPTEMBER 25, 1920. PRICE TWOPENCE

### SHOULD VOLUNTARY AND POOR-LAW HOSPITALS COMBINE?

We think it was of the London Hospital and an adjoining Poor-law institution that it was said that, while at the former every bed was being fully utilised, and in addition a large number of carefully chosen cases were waiting for admission, at the latter many wards were empty and the large standing charges, which should have been distributed over many patients, were being incurred in respect of a comparative few. The germ of the idea, therefore, of voluntary hospitals and Poor-law institutions working together originated long before the present campaign of Sir Arthur Stanley and Sir Napier Burnett in preparation for Our Day of the British Red Cross Society and Order of St. John of Jerusalem, which is to be held on October 4. In preparation for this event many interesting things are being said about hospitals and the hospital crisis, and one of the most interesting is a suggestion made by Sir Napier Burnett that the situation, as it refers to the long waiting-lists to be found in connection with most voluntary hospitals, might be met by Poor-law hospital buildings being handed over to voluntary committees for administration as voluntary hospitals.

We have heard a great deal in the past about voluntary hospitals being handed over to the State or to municipal authorities, but the present proposal, so boldly expressed, is, indeed, the other side of the argument, and when it is supported by an opinion from the same reliable source that if hospitals were municipalised they would cost, at a very moderate estimate, one-third more than at present, the suggestion of combination on these new lines is well worthy of the attention of the ratepayer. Certainly, so far as tradition goes, the voluntary hospital service is the senior service, and it has been built up during several centuries out of the experience and wisdom of the leading medical and lay thinkers of the community, and in its present form it has the great advantage of being able to learn not only from past mistakes, but from those unusual periods of stress in respect of war, famine, and pestilence through which the nation has passed during the history of voluntary hospitals. On the other hand, the municipal hospital is a thing of yesterday, and the only point about it that has yet been clearly brought home to the ratepaying public is its costliness and want of economy in dealing with the sick poor.

In regard to the very topical question of State or municipal help, we are glad to see that the line taken by the Red Cross propagandists is rather in the direction of subsidy by the State than what is likely to be more troublesome to the voluntary hospital service—namely, subsidy by the municipality or payments made by them in respect of patients, on the lines followed by Dr. Addison's Miscellaneous Powers Bill. At a meeting addressed by Sir Napier Burnett in Birmingham, a report of which will be found on page 652, in a speech bristling with inspiring points, we see that he expressed the opinion that the wife and family, the employer, the insurance companies, the State and municipality, reap great benefits from the existence of hospitals, to the maintenance of which, however, they do not adequately contribute. The result is a burden on voluntary effort which it is not meant to bear. Hospitals, he considered, should be subsidised by the State in consideration of what they do for the national welfare.

We have on many occasions pointed out how heavily the hospitals have been exploited under the National Health Acts, and it is likely that further great drafts have been made upon their resources, to the benefit of employers and employees and litigants under the Employers' Liability Acts. It should not be an enormous task to bring home to those who should pay in these respects their responsibility for the cost that improperly falls upon the hospitals. With regard, however, to the calls that are now being made upon the patient and the patients' friends, it appears to us that this source of revenue is strictly limited, and that, if collected with any amount of rigour, it will defeat its own ends by keeping beds waiting empty during negotiations, thus wastefully utilising the main machinery of the institution, with the effect of an increase in the average cost per bed. We do not at this juncture wish to take in fewer patients; rather do we desire to take in more, and thus extinguish that sinister feature which has constantly of late years been the skeleton in the cupboard of the voluntary system—namely, the waiting-list.

It is clear also, from the signs of the times, that the wave of universal but spurious prosperity which has been passing over the nation since the Armistice is on the point of subsiding. It is likely that, even though wages may remain high, there will not be so many people earning them. In many directions—in the cotton industry, in the automobile trade, in manufactories of boots, shoes, linen, hosiery, and woollen goods—trade is on the decline, and the slump in one trade naturally affects a number of other trades. If things continue to move in the direction in which they appear to be going, revenue from patients' payments, from employees' collections, and from employers, will be seriously affected. We do not say these things in an unduly pessimistic spirit, but merely to suggest what appears to us to be a necessary warning against hospitals relying too much on this or that source of revenue. We must, it appears to us, rely rather on a wider basis, such as that suggested by Sir Napier Burnett, and if those who are wisest in determining the destinies of the voluntary system decide for State subsidy we must cheerfully accept State subsidy, together with any drawbacks and any advantages that it brings with it.

